# The 8th Symposium on Global Cancer Research: Recognizing Creativity and Collaboration to Support Global Cancer Research and Control

**DOI:** 10.1200/GO.20.00199

**Published:** 2020-07-27

**Authors:** Linsey Eldridge, Mishka K. Cira, Kalina Duncan, Paul Pearlman, Satish Gopal

**Affiliations:** ^1^ National Cancer Institute Center for Global Health, Rockville, MD; ^2^ Clinical Monitoring Research Program Directorate, Frederick National Laboratory for Cancer Research, Rockville, MD

The global burden of cancer continues to rise, with the number of new cases expected to grow from 18.1 million to 24.9 million by 2040.^1^ Although cancer presents a global challenge, low- and middle-income countries (LMICs) disproportionately shoulder the burden. It is estimated that more than 70% of cancer cases occur in LMICs,^2^ yet these countries receive only 5% of the global share of resources for cancer care and control.^3^

The burden of cancer in LMICs demonstrates the importance of including cancer research and control in the broader global health dialogue. A collaboration between the Consortium of Universities for Global Health (CUGH) and the Center for Global Health (CGH) at the National Cancer Institute (NCI) grew out of a mutual recognition that the global cancer burden has been under-represented in the global health field and that addressing this burden requires creating opportunities for collaboration and the exchange of knowledge.

In 2013, CUGH and CGH partnered to launch a global cancer satellite meeting at the CUGH Annual Conference. At that time, an informal analysis of global activities of NCI-Designated Cancer Centers (NDCCs) revealed that several NDCCs were working in many of the same countries,^4^ some without knowledge of the work of other NDCCs. The NCI Cancer Centers Program was established as a part of the National Cancer Act of 1971 to recognize centers for their rigorous standards in transdisciplinary cancer research.^5^ Since 2013, what is now the Annual Symposium on Global Cancer Research (ASGCR) has become an enduring partnership between CUGH, CGH, NDCCs, ASCO, and the American Association for Cancer Research (AACR). CGH is interested in promoting the expansion of global oncology activities at NDCCs, so they, along with ASGCR, gather individuals to discuss trends and gaps in global cancer research and control and to map collaborative efforts that will help move the field forward.

The field of global oncology has grown remarkably over the past 8 years.^6^ One measure of this growth can be seen in the most recent (2018-2019) survey of NDCCs.^7^ Results from the survey, conducted with the ASCO Academic Global Oncology Task Force, revealed that 98% of NDCCs participate in global oncology activities. By comparison, 81% of NDCCs reported global oncology activities when previously surveyed in 2012.^2^

The 8th ASGCR was a collaboration with the Georgetown Lombardi Comprehensive Cancer Center, the Sidney Kimmel Comprehensive Cancer Center at Johns Hopkins University, the University of Maryland Marlene and Stewart Greenebaum Comprehensive Cancer Center, CUGH, ASCO, and AACR. The 2020 Symposium theme, Creative Approaches to Global Cancer Research and Control, recognized that sustainable research and control in low- or high-resource settings requires creativity and collaboration. As with the NDCC survey, increasing momentum for global oncology as an emerging academic discipline was again evident in preparation for the 8th ASGCR, which fielded a record number of abstract submissions (180 abstracts from 38 countries) and registrations (reaching capacity 8 weeks before the meeting) compared with previous years. Originally scheduled for April 2020, the meeting was ultimately cancelled because of the ongoing COVID-19 pandemic, guidance from the US National Institutes of Health (NIH), and in coordination with the CUGH Conference organizers.

Despite this unfortunate cancellation, we are excited to acknowledge the work put forth by the global oncology community this past year. In addition to this special *JCO Global Oncology* publication of accepted abstracts, top scoring authors will have the opportunity to present their abstracts virtually, and time-sensitive scientific content of the symposium will be shared via webinar in partnership with CUGH. This allows for the dissemination of cancer-related content to the broader global health community, despite disruptions to the in-person meeting from the COVID-19 pandemic.

The COVID-19 pandemic serves as an important reminder that addressing urgent public health challenges requires creativity and global collaboration. Established in 2011, CGH was created to coordinate and expand NCI global cancer research activities.^8^ CGH works through partnerships across the NCI, NIH, NDCCs, professional societies, and other governmental and nongovernmental organizations to convene the global cancer community, support global cancer research and training, and disseminate evidence that informs domestic and global cancer control efforts. In the past 10 years, CGH has supported—through funding or coordination—192 grants and supplements with collaborators from 62 countries, including 51 LMICs. These projects include international bilateral partnership programs,^9^ development of affordable technologies for cancer detection and treatment that can be implemented in LMIC settings,^10,11^ and initiatives to support NDCCs to conduct global cancer research to answer important scientific questions, improve research capacity, and generate evidence that advances global cancer control. CGH has also supported more than 150 trainees from more than 50 countries in global cancer research and control through programs like the Short-Term Scientist Exchange Program^12^ and the NCI Summer Curriculum for Cancer Prevention and Control.^13^

To amplify the impact of cancer research and training initiatives, the dissemination efforts of CGH are modeled on the US Cancer Control National Partnership, which is focused on applying evidence to inform comprehensive cancer control measures.^14^ Since 2013, more than 500 individuals from 59 countries have participated in CGH-supported dissemination activities. For example, the CGH Cancer Control ECHO Program links cancer researchers and program implementers in LMICs in a knowledge-sharing network to discuss existing and needed research that can inform cancer control initiatives.^15^ Findings from these partnerships are included in the abstracts in this issue of *JCO Global Oncology*. Today’s unprecedented awareness of the global cancer burden marks a timely opportunity for making measurable progress as a community toward global cancer control.^16^

As CGH and ASGCR look ahead to their second decade in existence and begin to plan the 2021 meeting, there are important opportunities to build on activities and successes to date. First, there continues to be a critical need to support research that addresses key scientific questions in global cancer control or that capitalizes on unique scientific opportunities afforded by global collaboration. Particularly in LMICs, it is common and understandable for research to be undervalued in the face of overwhelming programmatic needs. However, the 29% decline in the annual death rate from cancer in the United States between 1991 and 2017^17^ did not occur fortuitously, but rather as a result of strategic investments intended to generate new scientific knowledge for cancer control accompanied by robust efforts to have a translatable impact on patients and communities. Although much of this existing knowledge can be directly applied to LMICs, much of it cannot. Studying cancer directly in LMICs also presents an opportunity to gain new knowledge that can benefit the world. CGH is initiating new programs that address tobacco control among HIV-infected populations in parts of the world most affected by HIV and that encourage researchers in the field of domestic cancer health disparities to extend their activities to global populations.

Second, addressing cancer on a global scale requires a global cancer workforce. This includes not only essential staff for delivery of critical services, but also investigators who drive the science needed for achieving global cancer control with appropriate rigor. Too often in LMICs, immensely talented young investigators who may have returned home after studying abroad encounter huge clinical workloads and overwhelming administrative responsibilities without ongoing mentorship and career development infrastructure that can allow them to become international thought leaders. CGH hopes to partially address this through a new funding opportunity for international research training programs to support the career development of the next generation of global cancer investigators in the United States and LMICs.^18^

Third, existing cancer knowledge must be translated into global cancer control policies, programs, and interventions. Recent experience with COVID-19 has offered a sobering reminder about the critical importance of science-based global public health policies. CGH has partnered with the Union for International Cancer Control and other organizations to establish the International Cancer Control Partnership (ICCP) as a collaboration of governments, cancer centers, and nongovernmental organizations to encourage evidence-based cancer control and to provide access to technical assistance and tools for use in the development and implementation of cancer control plans.^19,20^

Finally, reducing cancer mortality in the United States has required robust multilateral partnerships, and CGH will continue to foster such partnerships for global cancer control, as is reflected in work from the 8th ASGCR highlighted in this issue. Global partnerships built on a spirit of collaboration and trust have achieved remarkable progress for addressing infectious diseases, including HIV, Ebola, and most recently, COVID-19, and can serve as a model for achieving similar, sustained progress to reduce cancer suffering worldwide.
